# Immunohistochemical Study of NR2C2, BTG2, TBX19, and CDK2 Expression in 31 Paired Primary/Recurrent Nonfunctioning Pituitary Adenomas

**DOI:** 10.1155/2019/5731639

**Published:** 2019-05-16

**Authors:** Xiaohui Yao, Yazhuo Zhang, Lijuan Wu, Rui Cheng, Chuzhong Li, Chongxiao Qu, Hongming Ji

**Affiliations:** ^1^Shanxi Provincial People's Hospital, Taiyuan, Shanxi Province, China; ^2^Key Laboratory of Central Nervous System Injury Research, Beijing Neurosurgical Institute, Capital Medical University, Beijing, China

## Abstract

This study investigated potential markers for predicting nonfunctioning pituitary adenoma (NFPA) invasion and recurrence by high-throughput tissue microarray analyses. We retrospectively studied two groups of patients: 60 nonrecurrent NFPA cases that included noninvasion and invasion subtypes and 43 recurrent cases that included primary NFPA. A total of 31 paired patient samples were evaluated (12 patients with one surgery and 31 who had undergone two operations, with both tumors analyzed). Expressions of nuclear receptor subfamily 2 group C member 2 (NR2C2), B cell translocation gene 2, T-box-19 (TBX19), and cyclin-dependent kinase 2 (CDK2) in surgically resected specimens were assessed by immunohistochemistry. The relationships between marker expression and clinical characteristics including age, sex, tumor volume, and follow-up time were analyzed. Tumor volume and invasion as well as follow-up time were significantly associated with invasion and recurrence (P < 0.01). Of the 60 nonrecurrent samples, 15/41 and 13/19 showed high NR2C2 expression in the noninvasion and invasion groups, respectively (*χ*^2^ =5.287, P = 0.021). NR2C2 was also overexpressed in 43 primary recurrent cases (*χ*^2^ =5.433, P = 0.02), whereas CDK2 (*χ*^2^ = 11.242, P = 0.001) and TBX19 (*χ*^2^ = 4.875, P = 0.027) were downregulated. In the 31 paired samples, NR2C2 was more highly expressed in the recurrent as compared to the primary tumor. High NR2C2 expression was associated with NFPA invasion, recurrence, and progression, while TBX19 and CDK2 were associated with NFPA recurrence.

## 1. Introduction

Pituitary adenomas are common and benign intracranial neoplasms, of which 14%–28% are nonfunctioning pituitary adenomas (NFPAs) [1]. NFPA are the most common type of adenomas when taking into account only macroadenomas. Although they typically grow slowly, some have an aggressive character and invade adjacent dura mater and surrounding bones before reaching the cavernous sinuses. This makes complete resection difficult, leading to postoperative recurrence or regrowth. Even after complete or near-complete surgical resection, 12%–58% of NFPA patients experience regrowth within 5 years, which cannot be effectively controlled by available therapeutics [2–5]. In contrast to functioning adenomas for which several effective and relatively safe targeted pharmacological therapies have been developed, a specific medical treatment for NFPA is still lacking [6]. The new WHO classification underscores the adoption of a pituitary adenohypophyseal cell lineage as the main principle guiding the classification of adenomas. The main technique for tumor classification is immunohistochemistry with the combination of immunostains for the main pituitary hormones and, when required, pituitary transcription factors [7]. Therefore, in order to develop improved treatment strategies, appropriate biomarkers for NFPA behavior must be identified. Various markers for the aggressive behavior of NFPA have been reported, including altered expression of nuclear receptor superfamily 2 group C member 2 (NR2C2), B cell translocation gene 2 (BTG2), TBX19, and cyclin-dependent kinase 2 (CDK2), but the relationship between histopathological features of the disease and clinical outcome is unclear [8–11].

NR2C2 is extensively expressed in the ovary, testis, and brain [12] and regulates different aspects of neuronal development [13]; it also induces tumor initiation under the control of various modulators [14, 15]. BTG2 is a member of the BTG/TOB protein family and is encoded by an early growth response gene. Abnormal BTG2 expression has been implicated in tumor development and progression. T-box (TBX19) (also referred to as TPIT) is a transcription factor that is expressed in the pituitary gland; the TBX protein family plays important roles in early embryogenesis [16] and is associated with the development of various malignancies [17]. CDK2 is an enzyme that mediates the G1/S transition, and its dysregulation contributes to tumorigenesis. Human CDK2 is expressed in the pituitary pars intermedia, likely through a mechanism that is active in cells with elevated proliferative activity [18].

At present, predicting NFPA behavior such as progression and recurrence remains challenging. To this end, we investigated the correlation between NR2C2, BTG, TBX19, and CDK2 and NFPA invasiveness and recurrence by immunohistochemical and tissue microarray (TMA) analyses.

## 2. Materials and Methods

### 2.1. Patients

We retrospectively reviewed 134 NFPA cases including 60 nonrecurrent patients at Shanxi Province People's Hospital and 43 primary recurrent NFPA cases at the Beijing Tiantan Hospital of Capital Medical University; among them, paired samples were available for 31 patients (12 with one surgery and 31 who had undergone two operations, with both tumors analyzed). The patients were all diagnosed with NFPA according to the 2007 World Health Organization histological classification from January 2008 to December 2014 and underwent transsphenoidal or transcranial operation that was followed up with magnetic resonance imaging. Demographic information such as sex and age; clinical information including tumor invasion, size, and volume; and clinical symptoms such as headaches, diminution of visual acuity, and defective field of vision were recorded for each patient.

The patients were classified into two groups. The nonrecurrent group included noninvasive (Knosp classification I and II) and invasive (Knosp classification IIIb and IV) tumors [19]. The recurrent group included 43 primary recurrent tumors, of which 31 were paired (primary and recurrent tumors). The quality of 12 specimens from the second operation was too poor for TMA, so only 31 paired primary and recurrent tumor specimens were included in the analysis. Recurrence was defined as a new lesion after complete remission or enlargement of a residual tumor, as confirmed histologically by a neurosurgeon and two neuroradiologists who were blinded to patient information. It has been documented that 10-20% of completely resected tumors recur after 5-10 years and when residual tumor remains after surgery, the percentage can be up to 50% within 5-10 years [20–22]. Although it is difficult to define nonrecurrence, in our study, the patients who were followed up for more than 6 years where there was no recurrence were defined as nonrecurrence. Compared to recurrence, it hints that the tumor did not recur temporarily and it also indicates that the tumor progresses slowly. This study was approved by the institutional review board and patients provided written, informed consent for their participation.

### 2.2. TMA Construction

TMAs were prepared using the BOND-III fully automated array instrument (Leica Biosystems, Wetzlar, Germany) from three 2.0 mm in diameter core biopsies of a representative tumor which were randomly ordered on the array. The TMA was cut into 4 *μ*m sections on a microtome, placed in a water bath at 50°C, and then transferred to positively charged glass slides. The specimens were deparaffinized and rehydrated through a graded series of alcohol with water, dried at room temperature for 24–48 h, and stored at −80°C until use.

### 2.3. Immunohistochemistry

The content and quality of samples on the TMA slides were confirmed by hematoxylin and eosin staining. TMAs were processed for immunohistochemistry with the automatic array device. Other carcinoma and pituitary tissue specimens were used as positive and negative controls, respectively. NFPA samples were mostly gonadotrophic tumors but also included silent tumors of other lineages and occasionally null cell tumors. Therefore, we included antibodies against growth hormone, prolactin, thyroid-stimulating hormone, adrenocorticotropic hormone (ACTH), follicle-stimulating hormone, and luteinizing hormone in the immunohistochemical analysis along with antibodies against the following proteins: NR2C2 (1:600), with 15 min each of alkaline epitope retrieval (ER) and heat-induced epitope retrieval (HIRE); BTG2 (1:50), with 30 min of acid ER and 15 min of HIRE; TBX19 (8:1), with 30 min of alkaline ER and 15 min of HIRE; and CDK2 (1:600), with 20 min of alkaline ER and 15 min of HIRE. The primary antibodies were from Abcam (Cambridge, MA, USA) and were detected with the Bond Polymer Refine Detection kit (DS9800; Leica Biosystems, Wetzlar, Germany). Digital images were acquired using an Aperio AT2 scanner (Leica Biosystems). Slides were independently evaluated and scored by two neuropathologists who were blinded to group assignment. Differences in interpretation were resolved by consensus.

### 2.4. TMA Scanning and Image Analysis

Immunopositive cells in 1000 tumors were counted under a light microscope by two neuropathologists; the positively stained area was expressed relative to the whole adenoma area. Staining intensity was scored as 0 (negative), 1 (weak), 2 (moderate), or 3 (strong). Immunopositivity for each biomarker was scored according to the following semiquantitative scale: 0 (no reactivity), 1 (1%–25% of neoplastic cells are positive), 2 (26%–50% positive), 3 (51%–75% positive), and 4 (76%–100% positive). The 12 scores were summed for statistical analyses. Tumors with a final staining score ≥ 6 were considered as having high expression, whereas a score < 6 was considered as low expression.

### 2.5. Statistical Analysis

Statistical analysis was performed using SPSS v.20.0 software (IBM Inc., Armonk, NY, USA). Results for categorical variables are presented as proportions and frequencies. Differences in categorical variables among groups were analyzed with the *χ*^2^ test or Fisher's exact test. Descriptive statistics are presented as the mean ± standard deviation. Comparisons between groups were made with the unpaired two-sample* t*-test. Tumor volume is presented as a median value with a range. Comparisons between groups were made with the Mann-Whitney test. Statistical significance was defined as P < 0.05.

## 3. Results

### 3.1. Characteristics of the Study Cohort

Of the 134 NFPA cases included in the study, 60 were diagnosed as nonrecurrent and 43 were primary recurrent, and paired samples were available for 31 patients. An endoscopic endonasal transsphenoidal approach was used in 87 cases (64.9%), a microscopic transsphenoidal approach in 37 cases (27.6%), and a craniotomy in 10 cases (7.5%). Mean patient age was 47 years (range, 31–59.5 years), and there were 45 men and 58 women. Chief complaints at the time of presentation were visual disturbance (n = 66; 64.1%), headache (n = 54; 52.43%), and visual field deficits (n = 20; 19.4%). Up to the end of the follow-up period (December 31, 2014), 43 patients had experienced recurrence after surgery; the mean duration (± SD) of follow-up was 31.81 ± 16.04 months.

We evaluated the interdependence of clinical parameters in nonrecurrent and recurrent primary patients with the unpaired two-sample* t*-test, Mann-Whitney test, and *χ*^2^ test. Parameters related to prognosis were selected based on our clinical experience. No patients underwent adjuvant radiotherapy during follow-up. We found that tumor volume and maximum diameter were associated with invasion and recurrence (P < 0.01), whereas invasion, tumor volume and maximum diameter, and follow-up time were associated with recurrence (P < 0.01; Table 1). This indicated that large tumors are more likely to be invasive and to recur and that invasive tumors have a greater risk of recurrence. There were no differences with respect to sex or age among groups.

### 3.2. NR2C2, BTG2, CDK2, and TBX19 Expression and Correlation with Adenoma

**Figure 1:**
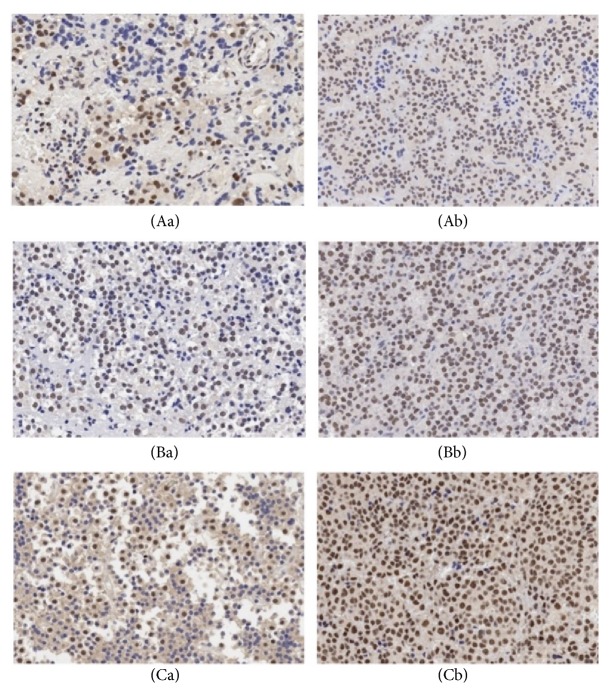
NR2C2 expressed in each group. (Aa): lowly expressed in noninvasive group; (Ab): highly expressed in invasive group; (Ba): lowly expressed in nonrecurrent group; (Bb): highly expressed in primary recurrent group; (Ca): lowly expressed in primary group; (Cb): highly expressed in recurrent group.

Of the 60 nonrecurrent samples, 15/41 and 13/19 showed high NR2C2 expression in the noninvasive and invasive NFPA groups, respectively (*χ*^2^ = 5.287, P = 0.021, Figure 1). CDK2 was upregulated, whereas BTG and TBX19 were downregulated in noninvasive and invasive groups, respectively. However, there were no differences in the levels of the three proteins between these two groups (Table 2).

**Figure 2:**
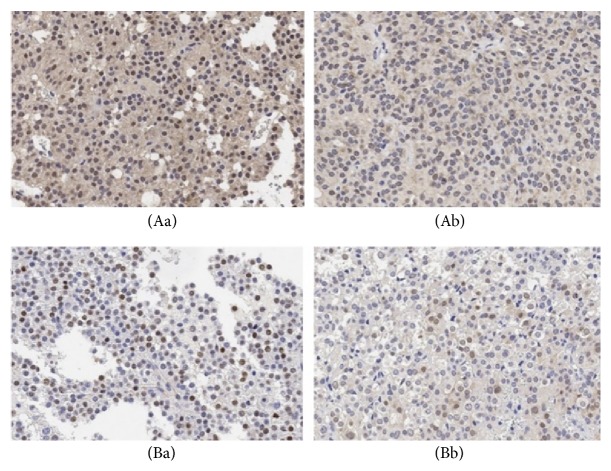
BTG2 and CDK2 expressed in each group. (Aa): BTG2 highly expressed in primary recurrent group; (Ab): BTG2 lowly expressed in nonrecurrent group. (Ba): CDK2 highly expressed in primary recurrent group; (Bb): CDK2 lowly expressed in nonrecurrent group.

Of the 43 cases of recurrent primary NFPA, 30 overexpressed NR2C2 as compared to 28/60 in the nonrecurrent group. NR2C2 was highly expressed in the primary recurrent group (*χ*^2^ = 5.433, P = 0.02, Figure 1). On the other hand, lowly expressed CDK2 was observed in 2/60 patients in the nonrecurrence group and in 11/32 patients in the primary recurrent group (*χ*^2^ = 11.242, P = 0.001, Figure 2). TBX19 was also expressed at a low level in 38/43 recurrent primary tumors (*χ*^2^ = 4.875, P = 0.027, Figure 2). BTG was also downregulated in nonrecurrent and primary recurrent NFPA, but there was no statistically significant difference between the two groups. Thus, primary recurrent tumors showed low expression of CDK2 and TBX19 but overexpression of NR2C2 (Table 2).

In 31 paired specimens, recurrent tumors showed higher NR2C2 expression than primary tumors (30/31 versus 24/31, *χ*^2^ = 4.167, P = 0.031; Figure 1). There were no differences in BTG, CDK2, and TBX19 expression between the two groups, although BTG and TBX19 were downregulated and CDK2 was upregulated. These results indicated that NR2C2 overexpression can be used to distinguish recurrent from primary NFPA tumors (Table 3).

## 4. Discussion

Surgery is the primary treatment in NFPAs. However, at least half of the cases result in subtotal or partial resection leading to significant rates of recurrence. Many unanswered questions remain regarding the management of recurrent NFPAs including surgical approach and medicine therapy [23]. Owing to its invasiveness and progression, pituitary adenoma—especially NFPA—is difficult to resect and treat. Recently, the term “pituitary neuroendocrine tumor” was proposed for pituitary adenoma to emphasize its unpredictable nature [24]. However, the relationship between molecular alterations and clinical outcome in pituitary disorders is not well understood. To address this issue, we examined the association between the expression of four biomarkers and tumor invasion and recurrence along with other clinical characteristics such as tumor diameter and volume and follow-up time. The results of our study showed that visual disturbances due to compression of the optic apparatus were common, as were headache and visual field deficits. Moreover, large tumors were more likely to be invasive and to recur, while the presence of invasive tumors and a longer follow-up time increased the risk of recurrence.

NR2C2, also known as testicular nuclear receptor 4, was first cloned from human hypothalamus, prostate, and testes [25]. NR2C2 plays an important role in the central nervous system, regulating many physiological and pathophysiological processes [26]. Phosphorylation and/or dephosphorylation, acetylation, cyclic AMP/protein kinase A signaling, a vitamin A metabolite, forkhead box O3a, and other factors were shown to suppress or enhance NR2C2 function in diverse biological processes [14]. NR2C2 can both inhibit and activate target genes depending on the physiological context [27]. For example, it was shown to stimulate the migration of liver cancer cells but suppressed liver cancer cell growth [28]. In breast cancer, NR2C2 interacts with estrogen receptor to inhibit MCF-7 cell proliferation [29]. NR2C2 is highly expressed in CD133+ prostate cancer stem/progenitor cells and promoted their invasion through enhancer of zeste homolog 2 signaling involving several metastasis-associated genes such as NOTCH1, SLUG, transforming growth factor *β*1, and matrix metalloproteinase 9 [30]. NR2C2 was overexpressed in pituitary adenoma and promoted ACTH secretion, cell proliferation, and tumor invasion [31]. In this study, we observed a clear association between elevated NR2C2 expression and tumor invasion and recurrence. Thus, NR2C2 plays an important role in NFPA invasion, development, and progression.

BTG2 functions as a tumor suppressor or promoter in various human malignancies [32]. Low BTG2 expression was linked to high tumor grade, size, and recurrence in estrogen receptor-positive breast cancer [33]. Additionally, BTG2 was downregulated in renal cell carcinoma as compared to normal control renal tissue, whereas BTG2 overexpression was associated with inhibition of cell growth, migration, and invasion [34]. A decrease in BTG2 level could indirectly activate AKT and extracellular signal-regulated kinase/mitogen-activated protein kinase signaling pathways and regulate the downstream effects of miR-25-3p to stimulate proliferation of triple-negative breast cancer cells [35]. Our results demonstrated that BTG2 expression was reduced in invasive, primary, and recurrent NFPA, suggesting a tumor-suppressor function.

TBX19 is involved in early embryogenesis, cell differentiation, and organogenesis of the pituitary gland and has also been implicated in carcinogenesis. TBX transcription factors are transcriptional activators or repressors [36]. TBX19 was shown to be more highly expressed in colorectal cancer tissue, which was associated with lymph node metastasis [37]. Meanwhile, it was also reported that TBX19 acts downstream of KRAS in human cancer [38]. TBX19 was the first TBX family member to be identified in the pituitary; it was shown to repress the gonadotrophic phenotype possibly through gonadotrophic-specific splicing factor 1 [36, 39] and thus modulate terminal differentiation of pituitary lineages. Specifically, TBX19 regulated the proopiomelanocortin lineage that gives rise to corticotropes. In the intermediate pituitary, TBX19 is essential for intermediate lobe and melanotrope differentiation and is expressed in paired-box 7-positive cells that are associated with suppression of (sex determining region Y)-box 2 and induction of differentiation. Thus, TBX19 is required for the terminal differentiation of pituitary proopiomelanocortin-expressing cells [40, 41]. In this study, TBX19 level was decreased in the primary recurrent group, suggesting a role in the recurrence of NFPA. However, given that it was also expressed at a low level in the invasion and recurrence groups, it could also act as a transcriptional repressor in the development of NFPA.

CDK2 regulates cell cycle progression and its activity depends on the binding of regulatory cyclin subunits. Inappropriate expression of CDK2 has been implicated in various malignancies including lung carcinoma, pancreatic carcinoma, ovarian carcinoma, and sarcomas [42]. CDK2 promoted lineage commitment of neuron-glial antigen 2-expressing progenitors in the adult subventricular zone and induced the differentiation of adult neural progenitor cells and oligodendroglia [43]. However, other studies have shown that CDK2 is not essential for neural progenitor cell proliferation and differentiation and hippocampal granule neuron survival* in vivo* [44], implying that CDK2 activity is context-specific. Low-to-moderate CDK2 expression is related to the risk of colon cancer, although CDK2 activity above a certain threshold may also pose a risk. Some cancers may transition through a low-CDK2 state at early, possibly benign periods of growth. In support of this “two-state” hypothesis, it was reported that only complete ablation of CDK2 blocked tumorigenesis [45]. We found here that CDK2 was downregulated in the primary NFPA group, suggesting that this is a marker for NFPA development; on the other hand, CDK2 expression was elevated in the invasion and recurrence groups, implying that a high CDK2 level contributes to tumor progression. This dichotomous role makes NFPA an attractive therapeutic target for NFPA treatment.

## 5. Conclusions

In this study, we found that NR2C2 expression is associated with the invasion, recurrence, and progression of NFPA. TBX19 and CDK2 were also found to be involved in NFPA recurrence. This suggests that therapeutic strategies targeting NR2C2 can suppress NFPA development, whereas those targeting TBX19 and CDK2 may prevent its regrowth. However, whether these three factors interact to promote NFPA and the detailed mechanism of this interaction remains to be elucidated. More work is needed to determine whether these factors interact to promote other types of pituitary adenoma.

## Figures and Tables

**Table 1:** Characteristics of the cohort.

	Noninvasion N=41	Invasion N=19	P	Nonrecurrence N=60	Recurrence N=43	P	Paired-recurrence N =31
Age	52.00±10.20	54.05±11.34	0.488	52.65±10.54	40.84±12.93	<0.001	37.48±11.58
Sex (M/F)	25/16	6/13	0.034	31/29	14/29	0.054	11/20
Volume	3591 (1680-6292)	8120 (5288-12818)	<0.001	4885 (2078-8085)	7936 (5240-14548)	0.001	10140 (6125-14548)
Maximum diameter	22.83±5.73	32.00±9.64	0.001	25.73±8.31	31.05±9.69	0.004	31.94±9.73
Invasion (N/Y)	-	-	-	41/19	17/26	0.004	12/19
Follow-up time	44.41±17.93	37.26±16.98	0.150	42.15±17.81	31.81±16.04	0.003	31.65±15.80

**Table 2:** Noninvasion group versus invasion group and nonrecurrent group versus primary recurrent group.

		Noninvasion	Invasion	X^2^	P	Nonrecurrence	Primary recurrence	X^2^	P
NR2C2	Low-	26(63.4%)	6(31.6%)	5.287	0.021	32(53.3%)	13(30.2%)	5.433	0.020
	High-	15(36.6%)	13(68.4%)			28(46.7%)	30(69.8%)		
BTG2	Low-	35(85.4%)	18(94.7%)	0.384	0.536	53(88.3%)	43(100%)	3.698	0.054
	High-	6(14.6%)	1(5.3%)			7(11.7%)	0(0%)		
CDK2	Low-	1(2.4%)	1(5.3%)	-	0.537	2(3.3%)	11(25.6%)	11.242	0.001
	High-	40(97.6%)	18(94.7%)			58(96.7%)	32(74.4%)		
TBX19	Low-	31(75.6%)	11(57.9%)	1.940	0.164	42(70.0%)	38(88.4%)	4.875	0.027
	High-	10(24.4%)	8(42.1%)			18(30.0%)	5(11.6%)		

**Table 3:** The paired recurrent group.

		Recurrence			
Primary		Low-	High-	X^2^	P

NR2C2	Low-	1(14.3%)	6(85.7%)	4.167	0.031
	High-	0(0%)	24(100%)		
BTG2	Low-	31	0	-	-
	High-	0	0		
CDK2	Low-	7(77.8%)	2(22.2%)	-	1.000
	High-	1(4.5%)	21(95.5%)		
TBX19	Low-	20(71.4%)	8(28.6%)	2.500	0.109
	High-	2(66.7%)	1(33.3%)		

## Data Availability

The analyzed datasets generated during the study are available from the corresponding author upon reasonable request.
